# Symptomatology of the Pupil

**Published:** 1882-06

**Authors:** Lucien Howe


### BUFFALO

### Medical and Surgical Journal.

Vol. XXI. JUNE, 1882. No. 11.

### Original Communications.

### Symptomatology of the Pupil.

By Lucien Howe, M. D.

PART II.—PATHOLOGICAL CHANGES.

In the first part of this article a short review was given of the leading facts relating to the anatomy of the iris and to its physiological movements. The general course of the nerves which supply it, was traced, and we found, that while sensitive branches of the trigeminus supplied its entire structure, its movements were regulated by two other nerves, distributed to two different portions. We learned that the circular band of muscular fibers surrounding the pupillary margin, was supplied by the motor oculi, and the radiating fibers by the sympathetic.

It was, hence, easy to perceive that a small pupil could result from strong contraction of the sphincter, or from unusual relaxation of the radiating fibers; and a large pupil could result from strong contraction of the radiating, or from unusual relaxation of the sphincter fibers. Bearing in mind this important connection, and also remembering how the pupil is contracted physiologically in old age, in the effort at accommodation for a near point, and under the influence of light, we are now prepared to advance to the pathological aspects of the question.

But here especially are we apt to become confused in searching for the origin of the unnatural size of a pupil, as there are, as just stated, two distinct causes which can produce the same result. For example, in a given case, the physician is in doubt whether the small pupil, which he sees in his patient, is due to some irritation of the motor oculi, thus contracting the sphincter of the iris, or due to a paralysis of the sympathetic, thus relaxing the radiating fibers.

All such cases would indeed prove exceedingly perplexing, were it not for our knowledge of a few facts in regard to the motor oculi. We know it is principally this nerve which causes contraction of the sphincter when light enters the eye, and in the act of accommodation, and also know that by the instillation of the extract of Calabar bean, this nerve is stimulated and the sphincter contracted; and finally, by a similar use of atropine* the opposite effect is produced. These facts can be utilized to advantage clinically. For example, in a case where the myosis is due to total paralysis of the sympathetic, even a still greater contraction of the pupil might be produced by the action of light, by the effort of accommodation or by the use of Calabar bean, by thus stimulating the motor oculi. But in such a case, if atropine be used, there is no dilatation beyond the usual limit, for, even though the sphincter be relaxed, the radiating fibers supplied by the already paralyzed sympathetic, have no power to contract. Should the repeated use of atropine in strong solutions—say three or four grains to the ounce—produce only moderate dilatation of the pupil, we may infer that with a relaxed sphincter the radiating fibers have contracted somewhat; in other words, that the sympathetic is only partly paralyzed.

On the other hand, if a dilatation be due to paralysis of the motor oculi, and consequent relaxation of the sphincter, it is impossible to produce contraction by the action of light, by an effort of the accommodation, or by using the extract of Calabar

* Atropine is here, and subsequently, used for that class of drugs, whose action is similar to that of Belladonna, and although "Calabar bean" is mentioned as producing contraction, it is ordinarily used in the form of eserine.

bean; moreover, atropine produces no effect. But if the mydriasis is caused by irritation of the sympathetic and consequent contraction of the radiating fibers, some change may be produced by the action-light, by the extract of Calabar bean, or possibly by the effort at accommodation. Stimulants of the motor oculi may, in such a case, cause even a greater degree of contraction of the sphincter than already existed in the radiating fibers, and therefore the pupil is made smaller. These are evidently important points of differential diagnosis.

In general, however, it may be said that concomitant symptoms must be relied upon in the formation of an opinion, and in all cases these furnish at least corroborative evidence. For example, if we find a contracted pupil with increased vascularity of certain parts of the head, or with any signs of disease in the neck or spinal cord, we may infer that a myosis is due to some imperfect action of the fibers of the sympathetic. On the other hand, if a dilated pupil is accompanied by a divergent squint, or by imperfect action of any of the muscles of the eye, supplied by the motor oculi, we are safe in saying that a mydriasis is due to more or less complete paralysis of that nerve, and, of course, to relaxation of the sphincter pupillæ.

In a paper of this kind it would of course be out of place, if not impossible to attempt to describe all the morbid conditions which are accompanied by peculiarities in the size of the pupil. It is only intended, at present, to state the general principles upon which such changes depend. Should any one be interested in a particular phase of the subject, he can gather information to better advantage with the aid of a list of references here appended.

It may be well, however, to simply call attention to a few diseases in which an altered size of the pupil is a more or less constant symptom. For the sake of convenient arrangement I will refer first to those which are accompanied by myosis, and then to those which are accompanied by mydriasis. But let it be understood, that these conditions are not necessarily constant and unvarying features throughout all the different stages of the same disease. For example, a morbid condition which is only sufficiently extensive to produce irritation of the motor oculi, would, as we have seen, be accompanied by a contracted pupil, but if the extent of that same lesion increased, it might be sufficient to produce a paralyzed state of the nerve, and would then be accompanied by a dilated pupil. With this general observation we may proceed to a consideration of some of the diseases accompanied by contracted pupils.

Let us therefore begin by turning our attention to

### I, SPASMODIC MYOSIS.

In this, the contracted pupil is due to irritation of the motor oculi. In such a condition, as already mentioned, the pupil may, perhaps, be still further contracted by the action of light, by accommodation, or by Calabar bean, but it should dilate under the influence of atropine.

The lesion is usually intercranial and frequently of an inflammatory type. Thus we find it in the stages of excitement, produced by large doses of alcohol, or as an effect of opium [2], of nicotine [114], and especially of Calabar bean [86]. Apparently opposite conditions may produce the same result as an irritant. Thus, Nothnagel [17] mentions that myosis may occur in anæmia of the brain, and also from sudden hæmorrhage into the Pons. In the early stage of meningitis [3, 10], the spasmodic contraction of the motor oculi is evinced not only by the contracted pupil, but by convergent strabismus.

In alcoholic amlylyopia we often see the pupil contracted, and the same condition exists in other sceptic conditions.

Inflammation of the cornea, sclerotic and iris, especially in their early stage, or when of a painful nature, are also occasionally accompanied by a contracted pupil.

An interesting illustration of contracted pupil is seen in the more advanced stages of the general paralysis of the insane. Through the kindness and assistance of Drs. Andrews and

Granger I have had an opportunity of examining the eyes of seven such cases at the Buffalo State Asylum for the Insane. The results are shown in the following table:

| Number. | Sex. | Initials of Names. | Age. | Stage of the Disease. | Eye affected. | Contraction by Action of Light. | Contraction on Accommodation. | Dilates with a four-grain Solution of Atropia |
|---|---|---|---|---|---|---|---|---|
| 1 | M. | N. R. | 38 | 2d. | Left. | None. | Slight. | Widely. |
| 2 | F. | A. M. | 40 | 2d. | Both. | Slight. | None. | " |
| 3 | M. | J. C. | 39 | 2d. | Left. | Slight. | Slight. | " |
| 4 | M. | P. D. | 67 | 2d. | Both. | Slight. | None. | " |
| 5 | M. | C. S. | 40 | 2d. | Right. | None. | Slight. | " |
| 6 | M. | F. D. | 39 | 3d. | Left. | None. | Slight. | " |
| 7 | M. | F. F. | 49 | 2d. | Right. | None. | Decided. | " |

From this limited number, and from other observations [32], it appears that in such cases further contraction does not occur regularly on attempting to accommodate, and exceptionally from the effect of light, but dilatation is invariably produced by a strong solution of atropine. One would naturally expect in this disease that even the nerve supply of the radiating fibers, also participating in the general decay, would become affected and the pupil refuse to dilate widely under atropine, but such is not the case.

Before leaving this variety of myosis, I would mention a case which seems of interest, as tending to show that syphilis may produce the condition under consideration. The patient, a woman of 27, contracted a chancre 15 years ago. She passed through the primary and secondary stages, developing most typical symptoms and about three years and a half ago began to complain of discomfort "when looking at anything near by." This gradually increased to continual pain, the pupils grew small, there was convergent strabismus and considerable photophobia.

When I first saw her at the Buffalo Eye and Ear Infirmary, on the 4th of September, 1878, both eyes were prominent and in each, the cornea rather small; anterior chamber was apparently narrow; pupil about the size of a small pin's head; with left, the vision was equal to fingers at 12 feet, and the right, with a plus 20 cylindrical glass, at 90° gave vision=$\frac{20}{50}$. Every effort at accommodation or the presence of light gave increased pain and exaggerated the existing strabismus. On the other hand a two-grain solution of atropine dilated the pupils widely.

This seemed to be, without doubt, a case of myosis due to irritation of the motor oculi and consequent contraction of the sphincter, as indicated not only by the behavior of the iris, but by the strabismus. As apparently a result of syphilis this is a condition, at least, quite rare[16].

We come next to consider that form of myosis, known as

II, PARALYTIC MYOSIS, caused by partial or complete paralysis of those branches of the sympathetic, which supply the radiating fibers of the iris. In this, as already stated, even greater myosis may be produced by the effect of light, by an effort of the accommodation or by Calabar bean, but atropine does not cause any very decided dilatation. With a partial paralysis of the sympathetic, some dilatation can, however, be produced by atropine. In these cases, if the paralysis of the dilating fibers is complete, then no abnormal enlargement of the pupil is possible, even supposing the sphincter be also relaxed. But in reality such total paralysis of the sympathetic or of any other nerve is exceptional.

In considering this form of myosis, it is well to keep in mind an anatomical fact, which it seemed best to mention first in this connection. Judging from dissections alone, one would suppose that the portion of the sympathetic, which supplies the radiating fibers of the iris, is connected only with the cavernous plexus. It is from that plexus that a twig goes to the ophthalmic ganglion, and thence to the iris. But various experiments [111, 112, 113, 101], and pathological conditions [56] show that there is a most intimate connection between these terminating fibers of the sympathetic and the upper part of the spinal cord. Some observers consider them as of spinal origin [28] and knowing also that the motor oculi is of cerebral origin, it is not unnatural that the two varieties of myosis and mydriasis should have been classified on this basis. Thus we may speak of a spasmodic cerebral myosis (the variety first mentioned) and a paralytic spinal myosis (that which we are about to consider); or, on the other hand, of spasmodic spinal mydriasis and a paralytic cerebral mydriasis [114].

A most striking example of the connection between the fibers of the sympathetic which supply the dilator of the iris, and the upper part of the spinal cord, is shown by the effects of injuries in that region, [24, 75, 57, 82, 73]. In these cases, irritation sometimes occurs primarily and with it, dilatation, but contraction of the pupil supervenes sooner or later—and often of the most extreme degree.

It seems probable that the partial myosis, produced by chloroform narcosis, is of a paralytic nature. Although a slight degree of dilatation may usher in the stage of excitement, yet contraction almost invariably occurs when the narcosis is complete, [92, 91, 89, 90, 53, 42, 108, 79]. Gradual dilatation accompanies a return to the stage of excitement, but when this shows itself suddenly in deep narcosis, when it is very decided, and is not preceded by any unusual irritation, it is an indication of extreme danger [77]. There are, however, occasionally slight variations of no importance, and it is therefore almost too much to say that a constant degree of myosis is an unfailing indication that the chloroform is being properly administered [30]. The subject offers an attractive field for further investigation.

Perhaps one of the most striking forms of this variety of myosis is seen in ataxia. By many it is called spinal myosis and to its various phases much study has been given [51 36]. As the posterior columns of the spinal cord are in this disease the principal seat of the lesion, it is quite certain that the small pupil is due to a paralytic affection of the sympathetic. In spinal affections, however, there is a peculiarity in the behavior of the iris, which might be considered almost as diagnostic, and it is, that while the pupil can be made still smaller by an effort of the accommodation, it does not, on the other hand, react as usual under the influence of light. In a similar manner does the pupil behave in the paralysis of the insane, except that it dilates with the use of atropine.

As a good example of paralysis of the sympathetic it may be of interest to cite a case which recently came under my observation.

The patient, J. M., was a laborer, 26 years old, who applied at the Buffalo Eye and Ear Infirmary on the 14th of last April. He had always enjoyed good health, but nineteen days before, he began to suffer some slight pain in the region of the angle of the jaw, on the right side. Five days later, the pain grew more intense in the right ear and was followed by a purulent discharge. These symptoms, of course, pointed to a purulent inflammation of the middle ear, with perforation of the membrana tympani—a diagnosis, which further examination proved to be correct. But this was the least interesting feature of the case. On viewing the patient's face, first from one side, and then from the other, it presented two aspects, almost entirely distinct. The right half was flushed and constantly moist with perspiration; the left, quite pale and rather dry, while the median line of demarcation across the forehead and down the nose to the mouth was distinctly visible. The temperature on the right side was also perceptibly higher than on the left. As for the pupils, the right was hardly more than half the size of the left. The behavior of the iris and concomitant symptoms indicated a condition of paralysis or, at least, parasis of the sympathetic, which produced the myosis and also suggested interesting pathological questions, which it is beyond the scope of this paper to consider. The case must be left with simply the reference to the peculiarity of the pupil and conditions accompanying it. Such typical examples are not common [39].

Passing next to the consideration of mydriasis, let us give our attention first to the variety known as

III, SPASMODIC MYDRIASIS.

This, of course, is due to irritation of the sympathetic and consequent contraction of the radiating fibers of the iris. In its most typical form some slight contraction may be produced by the action of light, by efforts at accommodation or by the use of Calabar bean. In other words, these stimulants to the motor oculi may counterbalance the already existing irritation to the sympathetic. Moreover, greater mydriasis is often obtained by the use of atropine. When the enlarged pupil is due to simple paresis of the sympathetic, as most frequently happens, then the effect of these experiments of course more nearly approaches what we should expect in the normal condition [54].

Examples of this variety are comparatively rare. Spasmodic mydriasis, however, occasionslly occurs when other muscles of the body are suddenly made tense, as, for example, a case is cited [78] of a soldier who could dilate the pupils by making his muscles rigid when standing "attention." The same symptom is met with in some cases of chorea [26].

It is not strange that disturbances in organs in distant portions of the body should, by irritation of the sympathetic, produce dilatation of the pupil. I have once seen a case in which monolateral mydriasis accompanied dysmenorrhœa. The connection between the two was too intimate to be considered simply as a coincidence.

The patent, E. H., was 20 years old, with mydriasis in the right, which had shown itself at intervals for a year, previous to October 28, 1876, the date of her first visit. Then the right pupil was double the size of the left. Reaction to light, or on accommodation was slight, but forcible contraction could be produced by extract of Calabar bean. In a few days, however, the mydriasis disappeared, but began to show itself again at almost regular intervals of four weeks, each time, lasting three or four days. On enquiry it was found that the dilatation was simultaneous with the begining of a menstrual period. Having been advised to consult her family physician concerning a dysmenorrhœa from which she suffered, I learned subsequently that when improvement in this respect occurred the mydriasis also disappeared.

Finally we have to consider the second form of dilated pupil or that known as

IV, PARALYTIC MYDRIASIS.

In this the sphincter of the iris is relaxed as a result of innervation of the motor oculi. With extreme cases the action of light or efforts at accommodation produce little or no change, and atropine is likewise powerless to increase the abnormal condition.

This form of mydriasis is common to the more advanced stages of inflammatory conditions which affect the motor oculi either at its origin or in its course [27]. Thus, it is seen in well-established forms of inflammation about the cerebellum, in the second stage of meningitis [4, 10], in chronic hydrocephalus, in epilepsy [4]; where there has been effusion of blood or serum in the brain sufficient to cause compression [10], or where an intercranial tumor [19] exists.

The imperfect effect of belladonna, or its alkaloid, in increasing the mydriasis of this variety, has been already stated [26 74], and that of Duboisia [25] is probably similar. In morbus Basedowii there is very seldom any change in the size of the pupil—in spite of the fact that the sympathetic is evidently involved—and the mydriasis, which has been noticed, is considered by Stellwag [25] to be due to paralysis of the pupillary branch of the motor oculi.

Far-sighted persons, being obliged to contract the sphincter of the iris more than others, in the effort of accommodation, it sometimes happens that that muscle become relaxed—fatigued as it were—and a paralytic mydriasis results. In two typical cases of this kind I have found that a suitable convex glass relieved the difficulty.

Paralytic mydriasis may also be produced by some distinct lesion in the eye itself. At least it is well stablished that certain abnormal conditions of the globe are accompanied by mydriasis and it is probable that this is of the variety now under consideration. It would seem that affections of the choroid are especially apt to be accompanied by a dilated pupil, and in this connection it may be well to refer briefly to a peculiar case illustrating the point.

On March 1, 1882, I was consulted by S. L., a laborer, 52 years old, who received a blow on the left eye about eight years before. Since then it became inflamed at intervals, giving rise to considerable pain. When first seen, the globe was rather prominent, there was slight ciliary injection, cornea clear, the anterior chamber wide, and the pupil so widely dilated that only a small rim of iris could, with some difficulty, be discovered. No sign of contraction could be produced by action of light, by effort at accommodation or by esserne. Although the lens was clear, the fundus could not be seen on ophthalmoscopic examination. The vision was reduced to imperfect perception of light, the globe extremely hard, and as the pain was constant and severe—only yielding to large doses of morphine—the wishes of the patient were acceded to, and the eye removed on the 10th of March. Subsequent examination showed that there had existed an inflammation of the choroid, and in one spot, which measured about 3 by 4 lines, this layer had become atrophied, leaving the retina in contact with the sclerotic, and these two coatings had there bent outwards into a staphalomatous projection.

It is beyond the scope of this paper to enter into the treatment of either myosis or mydriasis, but a word or two concerning them may not be out of place. Being ordinarily dependent upon a deep-seated cause, it is necessary first to determine that, before any plan of rational treatment can be decided upon. But it often happens that there is doubt as to the origin of the trouble, and the patient still anxiously asks for a prognosis based at least upon the general probabilities of his case. In some of these rather obscure cases, if we have to do with a contracted pupil, it is moderately safe to say that the prospects are bad. The causes upon which a well established myosis depend, are not such as ordinarily tend to improvement, or yield to treatment.

On the other hand, many cases of mydriasis do recover spontaneously and it would seem that the tendency to cure can be hastened. I have twice observed a dilated pupil become gradually and permanently smaller under the daily use of the constant current, or by the continued instillation of extract of Calabar bean, in solutions just strong enough to produce a return to the normal size. This observation has been made by other practitioners [29, 114, 41, 88].

Before dismissing the subject, it is necessary to call attention to some pcculiarities which are not referred to in the remarks already made. It is but fair, for example, to mention that instances are not rare in which the pupils are of unequal size in the two eyes—and perhaps have been so from childhood—although the person may be considered perfectly well in every respect. I can, at once, recall three such cases among my acquaintances. At least an inequality in the refraction of the two eyes is however usually found in these instances.

Finally, it should be noted that as certain causes may produce an inequality in the size of the pupils of the two eyes, so the same causes, acting in some obscure manner, may result in irregularity in the form of the pupil of one eye, or of both. It would be too great a digression to discuss here the varying circumstances which may produce this, but the general principles, already stated, will furnish indications of the cause in doubtful cases.

Again, as we have already seen, that the same disease may be accompanied by a small pupil at one stage, and later, by the opposite condition; so it may occur, that variations show themselves in quick succession and it may be difficult to determine whether if we have to do with a rapidly recurring myosis, or mydriasis, or both combined. This is especially the case in some attacks of hysteria [105] and it is possible that this is due to the principle of associated movements of muscles, as seen in other parts of the body. In a similar way, deep inspiration or severe dispnœa is often accompanied by dilatation of the pupil.

Enough has been already said to show that in any given case, when the physician attempts to form any opinion from the altered size of the pupil, he must keep clearly in mind the physiological causes upon which such changes depend. It is almost useless to attempt to remember that this disease is accompanied by a contracted pupil, and that one, by a dilated pupil. The only true method is pursued by the practitioner who, keeping in mind the physiological conditions, seeks for the cause of the abnormal appearance. It was with the hope of making that relation between health and disease a little more clear, that it has seemed worth while to review these facts and personal observations.

---

REFERENCES.

1—Ackroyd, W., On the Action of Light on the Iris. Proceed. of the Roy. Soc. of Edinburgh. X p. 37. 1878.

2—Alcock, Nathaniel, How Opium contracts the Pupil. *Med. Times*, vol. 40, p. 521. 1870.

3—Bristow, Theory and Practice of Medicine, pages 871 and 954.

4—Eulenburg, in Ziemssen's Cyclopædia, vol. XIV, p. 127.

5—Flint's Practice of Medicine, p. 669.

6—Gradle, H., The Movements and Innervation of the Iris. *Chicago Journal of Nervous and Mental Diseases*, April 1875, p. 192, and July, 1875, p. 317.

7—Harlan, George C., Contraction of Pupil, with partial Paralysis of Accommodation.—*Medical Times*, May 1, 1871.

8—Hutchinson, J., Myosis and Iridoplegia from Brain Disease. Autopsy—Ophth. Hosp. Rep. VII, p. 39. 1871.

9—Hutchinson, S., Symptomatology of the Pupil. Brain, I. I. 1878.

10—Haguenin, in Ziemssen's Cyclopædia, vol. XII, pages 419—667.

11—Jackson, Hughlings. Conditions of the Pupil in Cases of Disease of the Nervous System. *Brit. Med. Journal*, April 6, p. 368.

12—Juaffreson, On a Case of Mydriasis with Paralysis of Accommodation. *Med. Times and Gazette*, No. 47, p. 89. 1873.

13—Keen, W. W., Tetanus treated with large doses of Calabar Bean without noticable effect on the Pupil—Cure. *Phil. Med. Times*, I. II. March, 1871, p. 195.

14—Le Conte, J., Adjustments of the Eye. *Amer. Journ. of Science and Arts*, Ser. II, vol. 47, 1872, p. 68—77.

15—Miller, M. N., A Case of Mydriasis, or Permanent Dilatation of the Pupil. *Med. Record*, Sept. 16, 1872.

16—Merie, Cases of Syphilitic Affections of the Third Nerve, producing Mydriasis with and without Ptosis. *Brit. Med. Journ.*, I., p. 29, 52. 1870.

17—Nothnagel, in Ziemssen's Cyclopædia, vol. XII, pages 34, 102, 139.

18—Nunneley, B., The Physiological Action of Atropine; Proceedings of the Royal Society. Vol. 18, p. 46. 1870.

19—Obernier, in Ziemssen's Cyclopædia, vol. XII, p. 250.

20—Pearse, J., On the Action of Hyoscymine and its Resemblance to Atropine. *Lancet*. 1876, p. 319.

21—Ringer, S., and Murell W., On Gelseminum Semper-virens. *Lancet*, 1876, I, p. 732 and II, p. 78.

22—Robertson, Argyll, On the Physiology of the Iris. *Lancet*, I. p. 211–212. 1870.

23—Russell, Three Cases in which Contraction of the Pupil was a prominent Symptom. *Med. Times and Gazette*, vol. 41, p. 392.

24—Russell, Displacement of the Seventh Cervical Vertebra; Injury to the Cord; Extreme Contraction of the Pupils. *Med. Times and Gazette*, vol. 41, p. 64.

25—Stellwag, Diseases of the Eye.

26—Von Ziemssen—Vol. XIV, p. 437.

27—Wells, Diseases of the Eye.

28—Walton Haynes, Diseases of the Eye, p. 321.

29—Webster, W. W., Two Cases of Mydriasis with Paralysis of Accommodation, treated by electricity. *Philadelphia Med. Times*, Oct. 24, 1874.

30—Winslow, W. H., Chloroform and the Pupil. *Philadelphia Med. Times*, vol. VI, 1876, p. 270.

32—Williams, Chas., Notes of Changes seen in the Eyes of ten Cases of General Paresis of the Insane. *Boston Medical and Surgical Journal*, Jan. 13, 1881.

---

32—Adamuek und Woinow, Ueber die Pupillenveraenderung bei der Accommodation. *Archiv f. Ophth.*, 1871, XVII, 1. S., 158–168.

33—Argyropulos, H., Beitraege zur Physiologie der Pupillarnerven. Inang. Diss., Giessen, 1878.

34—Baumeister, E., Direkte Reaction der einzelnen Pupillen auf Licht bei angeborener Amaurose. *Archiv f. Ophth.*, XIX, 2. S. 272.

36—Bergh, A., Fall von Myosis Spinalis. *Hygiea*, 1876.

37—Bessau, G., Die Pupillenenge im Schlafe und bei Rueckenmarkskrankheiten. Inaug. Diss., Koenigsberg, 48. 1879.

38—Brucke, Beschreibung des menschlichen Augapfels. 1847.

39—Chvostek, Unilaterale Ephidrose.

40—Curschmann, Ueber Pilocarpen. *Berl. Klin. Wochenschrift*, Nr. 68 u. 71. 1877.

41—Dor, Ueber Behandlung der Mydriasis durch den inducirten Strom. 1875.

42—Fischer, George, Experimentalle Studien zur Therapeutischen Galvanisation des Sympathicus. *Archiv f. Klin. Med.*, XVII, p. 1. '74.

43—George B., Ein Beitrag zur Wirkung des Jaborandi auf den Sphincter Pupillæ und Accommodationsapparat. Inaug. diss. Greifswald. 1875.

44—Gerlach, J., Ueber die Beziehungen des ciliaren Ursprungs der Iris zu dem Brucke'schen Muskel. Sitzungsber. der Phys. Med. Societaet zu Erlangen. Heft II, S. 49.

45—Gruenhagen, Einfluss der Temperatur auf die Pupillenweite. *Berl. Klin. Wochenschrift*, p. 21 und p. 525.

46—Gruenhagen und Samkowy, Ueber das Verhalten isolirter glatter Muskeln bei elektrischer Reizung. Archiv fuer das ges. Physiol. X p. 165–171. Nachwort von Gruenaagen, p. 172–173.

47—Gruenhagen, Ueber den Einfluss der verschiedenen Temperaturgrade auf die Iris der Saeugethiere und auf die willkuerliche Muskulatur des Frosches. *Tageblatt der Wiesbadener. Naturforscherversammlung*, p. 69. 1873.

48—Gruenhagen, (*a*), Ueber Pupillenerweiternde Nervenfasern; (*b*), Ueber den cerebralen Verlauf der Pupillenerweiternde Nerven. *Berliner Klin. Wochenschrift*, 1879, page 407 und 649.

49—Gruenhagen, A., Zur Irisbewegung. *Archiv f. d. ges. Physiologie*, p. 440. 1870.

50—Hellman, M., Beitraege zur Kenntniss der physiologischen Wirkungen des Hyoscymins und der Spaltungproducte des Hyoscymins und des Atropins. *Inaug. Diss. Jena.* 1873.

51—Hempel, Ueber die Spinalmyosis. *Archiv f. Ophth.*, XXII, 1. p. 1-28.

52—Henle, Handbuch der Anatomie des Menschen, II., 1866, p. 634.

53—Hirschberg, Ueber den Einfluss der Chloroform-Narcose auf die Pupille. *Berl. Klin. Wochenschrift*, p. 652. 1876.

54—Hirschler, J., Zur Casuistik der Mydriasis Spastica. *Wiener Med. Wochenschrift*, 1873, p. 387-393.

55—Hurwitz, A., Ueber die Reflexdilatation der Pupille. *Inaug. Diss. Erlangen.*

56—Jany, Zwei Faelle von Functionsstoerung im Gebiete des Halssympathicus. *Berl. Klin. Wochenschr.*, 1874, p. 104.

57—Kaempf, Fall von Myosis Paralytica durch Verletzung des Sympathicus. *Allgem. Wiener Med. Zeitung*, p. 85. 1872.

58—Katyschen, J., Ueber die electrische Erregung der sympathischen Fasern und ueber den Einfluss electrischer Stroeme auf die Pupille beim Menschen. *Arch. f. Psych.*, VIII, p. 624. 1878.

59—Koch, W., Ueber das Chloroform und seine Anwendung in der Chirurgie. Vortrag No. 80, in der Volkman'schen Serie. 1874.

60—Krenchel, W., Ueber die Wirkung des Muscarin auf Accommodation und Pupille. *Arch. f. Ophth.*, XX. 1. p. 135-150.

61—Krenchel, Untersuchungen ueber die Folgen der Sehnervendurchschneidung beim Frosch. *Arch. f. Ophth.*, XX. 1. p. 127-134.

62—Kugel, L., Ueber den Einfluss des Krystallkoerpers auf die Spannung der Regenbogenhaut. *Arch. f. Ophth.*, 1871, XVI. 1. p. 328-335.

63—Leeser, J., Die Pupillarbewegung. Wiesbaden. 1881.

64—Liersch, Ueber die Zeichen des Todes am menschlichen Auge. 1873.

65—Loewe, L., Beitrage zur Anatomie des Auges. *Arch. f. Mikrosk. Anat.*, XV. 4, p. 542.

66—Marme, W., Ueber Duboisia Myopodioides. *Goetting. Nachr.*, S. 413. 1878.

67—Meyer, A., Die Nervenendigungen in der Iris. *Centralbl. f. d. Med. Wissensch.*, Nr. 7.

68—Panse, C. H., Ueber die Nerven der Iris. *Arch. f. Ophth.*, XXIII, 3. S. 1. und Inaug. Diss. Erlangen.

69—Papowa, Alexandrine, Uutersuchungen ueber die Wirkung des Physostigmin. *Inaug. Diss.*, Bern. 1877.

70—Rachlmann, E., und Witkowsky, L.. Ueber das Verhalten der Pupillen waehrend des Schlafes. *Arch. f. Anat. und Physiol.*, 1878. (Physiol. Abt.) S. 109.

71—Rembold, Ueber Pupillarbewegung. *Inaug. Dissert.*, Tubingen. 1877.

72—Rembold, S., Ein Fall von Chloroform Intoxication per Stomachum, nebst Bemerkungen ueber das Verhalten der Pupille in der Chloroform Narkose. Nagel, Tuebingen. 1879.

73—Riegel, Franz, Ein Fall von halbseitiger Verletzung des Rueckenmarks. *Berliner Klin. Wochenschrift*, 1873, p. 208-210.

74—Rossbach, M. J., und C. Froehlich, Untersuchungen ueber die physiologischen Wirkungen des Atropin und Physostigmin auf Pupille und Herz. Verhandlungen der physikal. med. Gesellschaft in Wuerzburg. Neue Folge, 1873, Band V, p. 1–79.

75—Reuling, Georg, Fall von Myosis durch linksseitige Laehmung der Pars Cervicalis Nervi Sympathici in Folge einer Schussverletzung. *Arch. f. Augen- und Ohrenh.*, IV, 1. 117–118.

76—Samelsohn, J., Ueber vosomotorische Stoerungen des Auges. *Arch. f. Ophth.*, XXI, 3, p. 29–99.

77—Schlaeger, H., Die Veraenderungen der Pupille in der Chloroform Narcose. *Centralblatt f. Chirurgie*, S. 385. 1877.

78—Schlesinger, Adolf, Eine Innervationserscheinung der Iris. *Pesther Med. Chirurg. Presse*, 13, p. 218. 1874.

79—Schlesinger, A., Totale Laehmung eines Theiles der Iris. *Szemeszet*, Beilage zum *Orvosi Heilth*, Nr. 2. 1874.

80—Vogel, Ueber die Veraenderungen der menschlichen Pupille bei der Chloroformnarkose. *Petersb. Med. Wochenschrift*, No. 13 und 14. 1879.

81—Scotti, C., Ueber die Wirkung des Pilocarpinum Muriaticum. *Berl. Klin. Wochenschrift*, Nr. 11. 1877.

82—Seeligmueller, Laehmung des Sympathicus neben Laehmung des Nervus Ulnaris durch Schussverletzung. *Berliner Klin. Wochenschr.*, p. 43.

83—Simonowitsch, Rosa, Ueber Hyoscyamin und dessen Bedeutung fuer die Augenheilkunde. *Arch. f. Augen- und Ohrenh.*, IV, 1. p. 1–45. Mit 6 Tafeln.

84—Stellwag von Carion, Ueber Atropin. (Klinischer Vortrag.) *Allg. Wiener Med. Zeitung*, 1872, p. 146, 154.

85—Surminsky, B., Ueber die Wirkungsweise des Nicotin und Atropin auf das Gefaessnervensystem und die Pupille. *Inaug. Diss.*, Erlangen. 45. S. 1877.

86—Von Boeck, in Ziemssens Cyclopædia, Band XVII, 702.

87—Wernicke, C., Das Verhalten der Pupillen bei Geisteskrankheiten. Virchow, *Arch. f. Path. Anat.* Band 56, p. 397–467. 1872.

---

88—Briere, Mydriase datant de 14 mois, guerie apres 10 jours de traitement. *Ann. d'Ocul.* T. 74, p. 84–90.

89—Budin, P., et Coyne, Recherches Cliniques et Experimentales sur l'etat de la Puppille pendant l'anesthesie chirurgicale produit par le chloroforme. *Arch. de Physiol. Norm. et Pathol.*, p. 61–100. 1875.

90—Budin, P., et Coyne, Des Phenomenes Pupillaires dans l'Asphyxie difference de ces Phenomenes dans l'Anesthesie chloroformique et dans l'Anesthesie Asphyxique. *Gaz. Med. de Paris*, 20 Fevr. p. 90. 1875.

91—Budin, De l'etat de la pupille pendant l'anesthesie chirurgicale produite par le chloroforme. Indications pratiques qui peuvent en resulter. *Gaz. des Hop.*, 1874, p. 910.

92—Budin, P., et Coyne, De l'etat de pupille pendant l'anesthesis chloroformique et chloralique et pendant les efforts de vomissements. *Gaz. Med. de Paris*, 20 Fevr., p. 90. 1875.

93—Corville et Rochefontaine, De l'ablation du ganglion premier thoracique du grand sympathique chez le chien. *Gaz. Med. de Paris*, Nr. 12. 1874.

94—Crequy, Action du jaborandi sur la vue. Soc. de Therap. *Bull. Gen. de Therap.*, T. 88, p. 428. 1870.

95—Debouzy, A., Considerations sur les mouvements de l'iris. 59 pp. Paris, A. Delahaye. 1873.

96—**Drouin, Alph.**, Note pour demontrer qu'it n'y a pas de rapport direct entre l'etat d'accommodation de l'oil et le diametre de la pupille. *Gaz. Med. de Paris*, Nr. 28.

97—**Drouin, Alph.**, De la pupille. Anatomie, phisiologie, semecologie. *These de Paris.* Paris, Delahaye, 389 pp. 1876.

98—**Dubujadoux, Paul**, Action de l'atropine sur l'iris et l'accommodation. *These de Doctorat*, Nr. 138. Paris, 1873.

99—**Fournet**, Mydriase du cote droit dans un cas d'hypertrophic des ganglions cervicaux du cote correspondant meningite spinale limitie a la region dorsale. *Recucil d'Ophth.*, 1879, p. 304.

100—**Franciel, Paul**, Essai sur les mouvement de l'iris. *These de Paris*, Nr. 249. 1874.

101—**Francois, Franck**, Sur le dedoublement du sympathique cervical et sur la dissociation des filets vasculaires et des filets irido dilatateurs, audessus du ganglion cervical superieur. *Compt. Rend.*, 1878. T. 87, 175.

102—**Francois, Frank**, Sur la dissociation des filets irido-dilatateurs et des nerfs vasculaires au-dessus du ganglion cervical superieur. *Progr. Med.*, Nr. 30. 1878.

103—**Francois, Frank**, Note sur les defaut de subordination des mouvements de la pupille aux modifications vasculaires. *Gaz. des Hop.*, p. 748. 1878.

104—**Galezowski**, Quelques considerations sur le myosis spontane. *Recueil d'Ophth.*, p. 174–178.

105—**Galezowski**, Contracture hystireque de l'iris et du muscle accommodative avec myopie consecutive. *Progr. Med.*, 1878, Nr. 3.

106—**Percepied, Elie**, De la mydriase. 80 pp. *These de Paris*. A. Delahaye. 1876.

107—**Roque, F.**, De l'inegalite des pupilles dans les affections unilaterales des diverse region du corps. *Arch. de Physiol. norm. et Pathol.*, 1872, N. 1, p. 48.

108—**Schiff, M.**, La pupille consideree comme esthesio-metre. Traduction de l'Italien par le docteur R. G. de Choisity, 35 pp. Paris, Bailliere et fils. 1875.

109—**Sichel**, Anomalie d'innervation de l'iris, etc. *Gaz. des Hop*,, p. 347. 1876.

110—**Sichel**, Anomalie d'innervation de l'iris. Paralysis du nerf de la sixieme paire du cote gauche, de cause specifique arec mydriase du meme cote. Guerison rapide. *Gaz. des Hop.*, Nr 44, p. 347.

111—**Vulpian, A.**, Note relative a l'influence de l'extirpation du ganglion cervical superior sur les mouvements de l'iris. *Arch. de Physiol. Norm. et Pathol. de Brown-Sequard.* Januvier. 1874.

112—**Vulpian, A.**, Sur les phenomenes orbito-oculaires produits chez les mammiferes par l'excitation du bout central du nerf sciatique. Apres l'excision du ganglion cervical superieur et du ganglion thoracique superieur. *Ebend.*, vol. 87, p. 231. 1878.

113—**Vulpian, A.**, Experience demontrant que les fibres nerveuses, dont l'excitation provoque la dilatation de la pupille, ne proviennent pas toutes du cordon cervical du grand sympathique. *Compt. Rend.*, vol. 86, p. 1436. 1878.

114—**Wecker, V.**, Valeur semiologique de la mydriase et de la myosis. *Gaz. des Hop.*, p. 141. 1879.

115—**Id.**, in Graefe und Saemisch, v. IV., p. 563.

---

116—**Albini, J.**, Rapporti tra i movimenti dell'iride e la funzione visira. *Il Morgagni, Gennajo*, p. 21–26. 1876.

117—**Reymond, C.**, Rivista sull'Atropino. *L'Osservatore.* 1870.

118—**Schiff, M.**, Anno sulle ricerche fatte nel laberatorio del museo di Firenze durante il 2 trimestre. 1872.

119—**Schiff, Maurizio e Pio Foa**, La pupilla come estesiometro. *Imparziale*, No. 7, Oct. 2, 17. Nov., 1874.

